# Development of a social capital scale for constructed families of gay, bisexual, and other men who have sex with men

**DOI:** 10.1371/journal.pone.0208781

**Published:** 2018-12-13

**Authors:** Meagan Zarwell, William T. Robinson

**Affiliations:** 1 LSU Health Sciences Center, School of Public Health, Behavioral and Community Health Sciences, New Orleans, Louisiana, United States of America; 2 NO/AIDS Task Force, d.b.a CrescentCare, New Orleans, Louisiana, United States of America; 3 Center for AIDS Intervention Research, Medical College of Wisconsin, Milwaukee, Wisconsin, United States of America; 4 Louisiana Office of Public Health, STD/HIV Program, New Orleans, Louisiana, United States of America; New York Blood Center, UNITED STATES

## Abstract

Despite previous empirical studies which have linked social capital to a number of health outcomes, few studies have investigated sub-group specific social capital among populations at increased risk for HIV infection such as gay, bisexual and other men who have sex with men (GBM). Many GBM of color belong to constructed families in which friends refer to each other with kinship terms such as parents and children. No studies have measured social capital provisions within constructed family networks. This study developed a preliminary instrument for assessing social capital among constructed families. The network level social capital scale incorporated the following theoretical domains hypothesized to define social capital derived from network membership: social influence, multiplex ties, heterogeneity, social cohesion, trust, quality of support, and compositional quality. A cross-sectional survey administered an eight-item scale to 131 GBM who belonged to constructed families. The factor structure and confirmatory factor analysis were assessed. Reliability was evaluated using Cronbach’s alpha to measure internal consistency. A final single factor solution was obtained which was comprised of six items with high factor loadings. The resulting measures were highly correlated with an alpha of 0.84 and each factor loading was well above 0.3. This study assessed the psychometric properties of a preliminary network level social capital instrument among GBM in constructed families. Future studies may utilize or adopt this scale to measure network-level social capital within other populations.

## Introduction

HIV continues to disproportionately impact specific populations despite public health approaches. Gay, bisexual and other men who have sex with men (GBM) are at elevated risk for HIV infection and account for more than half of all people living with HIV in the United States and approximately 70% of all new infections annually[1]. The disparities for GBM of color are striking: an estimated one in two black GBM and one in four Latino GBM will become infected with HIV in their lifetime if current rates persist[2]. Better strategies to engage GBM and GBM of color in HIV prevention are warranted.

One such avenue for network level intervention are constructed families of gay and bisexual men. Historically, constructed families are social connections that have served as important sources of identification and a foundation of support to LGBT persons[3]. Constructed families are comprised of biologically unrelated individuals, often GBM and transgender women, who refer to each other with kinship terms such as “parents” and “children” [3–7]. Membership in constructed family networks may buffer against stressors many LGBT people of color experience including multiple intersecting stigmas due to their sexual and racial identities and therefore may be important to leverage for HIV prevention and interventions.

The term constructed family encompasses membership in “gay families” as well as performance networks such as the house and ball community and pageant families [3–7]. Within the house ball community, “houses” refer to a family surname, often the same as well-known fashion designers, and members compete in performance categories at community thrown balls[8–12]. Pageant families are similar to houses because individual members may compete in gay pageant competitions; however, these competitions are not synonymous with balls and awards are judged using different beauty and talent categories. Pageant families may also share common identities by informally adopting a family surname. In comparison, gay families, while similar to both the house ball community and pageant families, are not always associated with performance or ball culture. Gay families frequently provide mentorship, social support, and resilience to LGBT people who experience adversity [13–17]. Gay families may also share a family surname but are not synonymous with performance culture. Researchers have argued that other forms of constructed families, including gay families, may be just as valid for HIV prevention interventions as membership in particular gay families can increase or decrease HIV risk[7,14,18].

While studies have examined HIV risk and prevention opportunities within the house and ball community in large cities, few studies have explored the larger context of constructed families, social connectivity to resources, and social capital [15]. While many studies have explored social support provided by constructed family membership[14,18,19], to date no studies have explored social capital attributed to membership in constructed families for gay, bisexual and other men who have sex with men. This study aims to develop a new measure of social capital among GBM who belong to constructed family networks.

The theory of social capital is predicated on the notion that social networks have value. A multivalent a construct, social capital has been applied to various physical and mental health outcomes and theorists have attempted to develop reliable and valid measurements at various levels[20,21]. Previous studies have investigated social capital as membership and participation in voluntary community organizations[22]; as collective norms, trust, reciprocity, and knowledge[23]; as social network ties, communication, and normative pressures that foster community resilience[24]; and as social support, social leverage, informal social control and neighborhood organization participation[25].

The last two decades have distilled two dominant social capital perspectives: the social network approach and the social cohesion approach [26]. These perspectives diverge in meaningful ways. First, the social cohesion approach refers to trust, reciprocity, and civic and social participation. In contrast, network approaches incorporate social network analysis methods to measure networks and the resources within them. Each approach shares an ecological perspective which acknowledges that social capital operates at multiple levels to influence individual-level health. The first perspective, influenced by Bourdieu, emphasizes the availability and access to resources for connected individuals within social networks [27]. Lin’s approach anchored social capital within social networks as a mechanism that allows individuals to attain goals and access embedded resources they provide [28–30]. Patterns of relationships have also been explored within networks to measure network structures and the presence of specific peers (i.e. alters) who provide specific ties[30]. Examples include name, position, and resource generators whereby a participant lists social network members and describes their roles and positions, prestige or status levels, and other specific resources [29]. In contrast, Putnam’s view, which popularized the social cohesion school, argues for measures of trust, norms, reciprocity, and sanctions[31,32]. Critics of Putnam’s social capital often suggest that the definition is inconsistent by calling into question the validity of its measures through time and space[20,25,30,33]. Regardless of approach, each dominant social capital perspective situates social capital as resources nested within a social group and the potential access to resources through membership in social networks.

Social capital has been associated with HIV-related outcomes in previous empirical research. Within the injection drug use literature, studies have explored social capital as resources resulting from affiliation within particular social networks whose structures or norms encourage risky injection behavior that may increase HIV risk [34]. Researchers have hypothesized that social capital, (defined as collective norms, trust, reciprocity, and knowledge about safe injection practices) within drug use networks may influence risk or protective behaviors in different contexts[23]. Recent studies in the US have linked measures of social capital to adherence to medication to treat HIV infections[35], HIV diagnosis[36], and as a predictor of gonorrhea, syphilis, chlamydia and AIDS cases[37]. Some international studies have measured social capital in relation to reduced HIV risk behaviors[38] and a decline in HIV incidence attributed to social norms within networks [39] within the general population. Social capital measured in terms of membership to voluntary community organizations has also been correlated with HIV risk behaviors [22].

Despite the historical foundation of social capital as a relational construct, few studies have employed network measures and social cohesion to measure social capital. Indeed, some have argued that network measures of social capital have been “lost in translation” in public health[40]. A small number of social capital studies have focused on social networks and community participation, however very few such studies are among GBM. One US study found that social networks influenced health behavior related to HIV risk and that risk perceptions varied by network position among adolescent females [41]. Social capital measured as community group participation in rural Zimbabwe indicated that women in networks with higher levels of participation had lower incidences of new HIV infection and higher rates of protective behaviors[42]. Another study found that social network ties, communication, and normative pressures reduced drug-related HIV risk among young adults by creating community resiliency in Bushwick, New York[24]. One notable study among GBM in Swaziland found that social capital, measured as social participation and social cohesion, was associated with increased HIV testing [43]. The current study further contributes to these prior studies and expands current knowledge of how social capital among GBM operates within constructed families.

The dearth of studies which examine social capital among gay, bisexual and other men who have sex with men and the disproportionate impact of HIV among GBM justifies new conceptualizations of the theory. If social capital is a collective construct created through participation in social organizations, then the construct may be characterized by aspects of the constructed family networks of GBM. We argue that a blended approach of social cohesion and social network approaches may be a better way to conceptualize social capital for GBM. The *social embeddedness* of constructed families likely indicates some caliber of social capital because members of are bonded through kinship terminology and maintain social ties.

During the unpublished formative research phase for the National HIV Behavioral Surveillance (NHBS) in New Orleans in 2014, GBM described strength of ties (including hierarchy, status), boundedness (names and group identity), and resources / social support (instrumental, emotional, and informational support) in relation to social group memberships in constructed families. The development of a scale for use among subgroups of GBMs supports the social-network based approach which reasons that social capital is at its core characterized by the boundedness of networks members[25]. From the perspective of the individual or ego, (i.e. the perspective of one individual within a social network), resources are exchanged and transactions occur through interaction with network alters (i.e. peers) [44,45]. Relatedly, boundedness refers to the degree to which networked members are defined on the basis of traditional group structures such as kinship, employment, or neighborhood. Constructed families of GBM represent each of these theoretical components of social capital as members provide resources from each other and are often bounded by a family name or identity.

**Fig 1:**
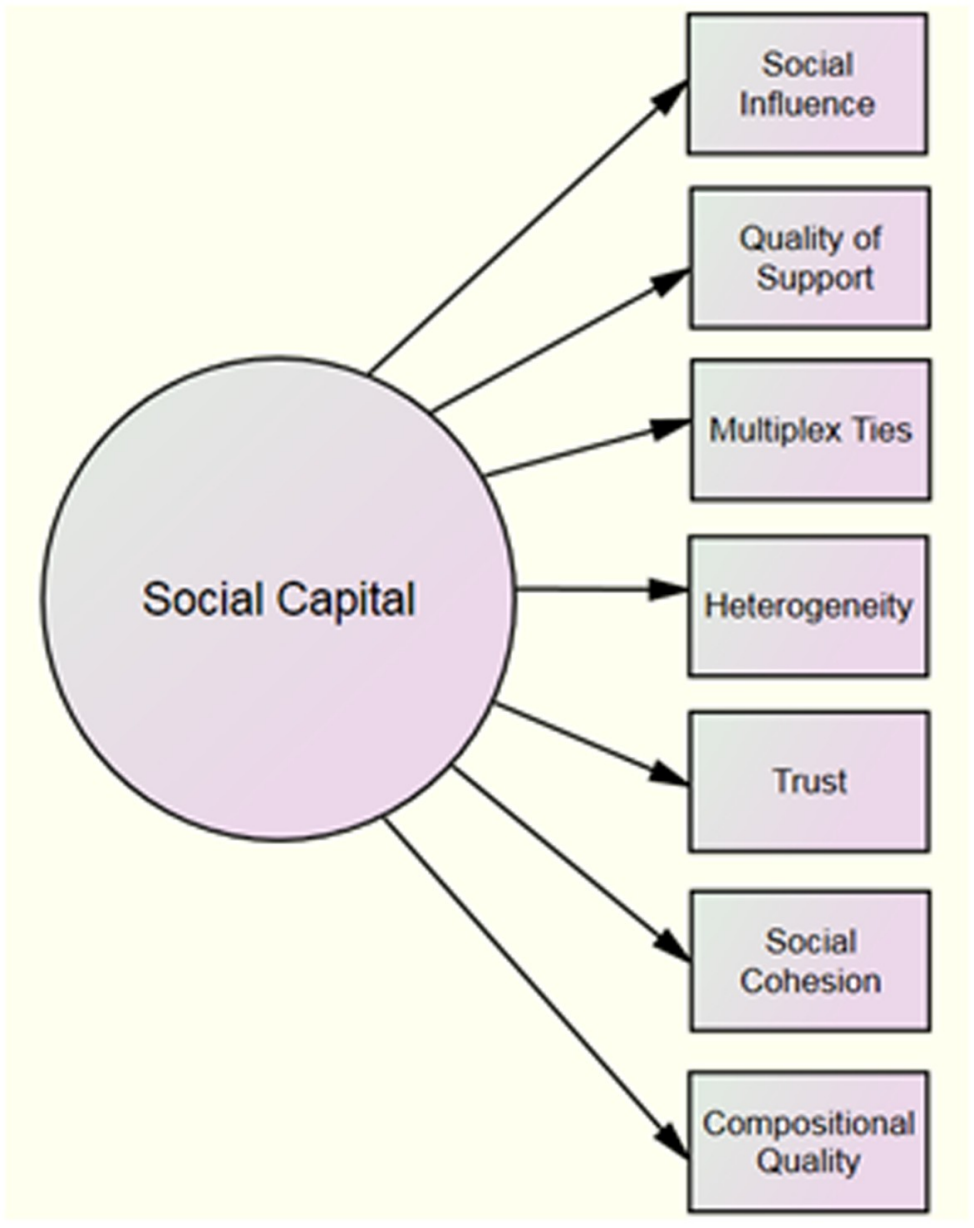
Social capital measures for use within constructed families.

Several network indicators were considered for the purpose of developing a measure of social capital within constructed families of GBM (see Fig 1). These measures were selected due to formative research in the community and upon close inspection of extant literature. S*ocial influence*, which measures the impact of network members on decisions made by ego, could be used to measure how individuals integrated within a family regulate behaviors of others. Evidence has shown that peer norms can influence health promoting behavior or activities that may be detrimental to health. In addition, forms of emotional, instrumental, or informational support are indicative of *tie strength*[33]. Social ties, including the number, quality, and content of constructed family members may foster healthy or risky behaviors among GBM. *Quality of support* may signify closeness among members that enhances social support[46]. Strong ties can be used to measure social regulation through a network. Whereas weak ties may link people across social groups, strong ties may require high relational support or restrict constructed family members from external information and resources[33]. *Multiplex ties*, another indicator of tie strength, exist in two or more relational contexts[44]. Within families, members who have multiple qualities may provide higher social support to individuals within families. Multiplex ties enhance social capital because stronger ties may result from connections that are multi-purposed (i.e. a friend and a coworker). *Heterogeneity* a measure that refers to diversity within networks is determined by the similarity of attributes of group members[33]. Indicators typically include socio-demographic variables such as gender, age or race. Belonging to a diverse constructed family may enhance resources available to ego. *Trust*, while a problematic indicator of individual level social capital, may be appropriate when used as aggregate to measure the trustworthiness in a group[47]. Perceptions about trust within a constructed family may indicate high or low social capital. *Social cohesion* is related to trust and refers to a contextual effect networks have on individuals that creates a sense of belonging and inclusion[26,48]. Thus, socially cohesive families may be those with the absence of covert conflict and the existence of strong social bonds. Finally, the *compositional quality* of a social network is indicated by the number of members with characteristics or resources deemed important by ego[33]. Thus, forms of support garnered from constructed family members may be beneficial to ego. Compositional quality may be measured by asking which network members are rallied for personal problems, financial support, or emotional wellbeing[32]. Specific qualities of constructed families may indicate social capital including having friends who help ego with personal problems, financial support, or emotional wellbeing. Relatedly, various types of social support garnered from constructed family members may improve social capital.

The purpose of this study is to assess social capital among gay and bisexual men in constructed families by developing a novel scale. Since studies have shown that social networks may be protective against HIV risk and the theory of social capital is a critical mechanism which mediates risky and protective sexual behaviors, it is hypothesized that social ties such as constructed families may promote varying levels of social capital and thus differences in health outcomes for GBM. **T**he significance of our focus on constructed families may benefit knowledge surrounding risk reduction and HIV prevention among younger GBM of color that experience multiple intersecting stigmas and experience the highest rates of HIV infection in the US. Moreover, this work contributes to the literature surrounding resilience and support cultivated among GBM despite the systemic oppression they may experience.

## Materials and methods

During the CDC’s National HIV Behavioral Surveillance (NHBS) MSM4 cycle in 2014 in New Orleans, eight items measuring network domains of social capital were asked to GBM who self-reported being members of constructed families: “*Please tell me whether you belong to any of the following groups*. *You can choose more than one option (Check all that apply)*.” The response categories included: gay family, pageant family, house ball community, faerie community, gay fraternity, bear community, leather community, other, and none. Anyone who reported belonging to a gay family, pageant family, or house ball community was considered a member of a constructed family. For the purposes of data collection, the term “gay family” was used throughout the interview because participants do not describe these relationships as “constructed families” and because participants frequently used the terms interchangeably (i.e. referring to their house as their gay family). In addition, we distinguished between constructed families with and without names because families with names were typically associated with performance. In addition, it was hypothesized that this distinction recognized a level of boundedness, prestige, or formality that may result from sharing a family name which may be an important distinction when measuring social capital.

### Participants

In accordance with CDC protocol, eligibility included cisgender men (i.e. born and self-identified male), aged 18 and older, who had ever had sex with a man, residents of New Orleans, and who were able to take the survey in English. Out of the entire sample of 553 participants, 131 reported belonging to a constructed family (CF). In addition to the standard core NHBS questionnaire, participants who reported belonging to CF (either a gay family, pageant family or house ball community) were asked a subsequent series of questions about membership.

### Recruitment

Trained interviewers recruited respondents through venue-based time-space sampling (VBTS) methods[49,50]. Briefly, GBM were systematically approached and screened for eligibility at venues such as bars and clubs in the metropolitan area. Formative research including observations identified venues and established day-time periods for recruitment of participants. A monthly calendar was used to schedule days and times for the recruitment events at venues in a two-stage sampling design[50]. Once selected, participants were asked to partake in an anonymous survey and HIV testing to protect privacy and prevent harm to participants. Interviewers conducted surveys on handheld computers. Participation was incentivized with a $25 cash-value gift card for the survey and an additional $25 cash-value gift card for HIV testing. All NHBS interviewers were trained and certified under the standards of the Louisiana Office of Public Health to provide a brief counseling session and test results to participants and maintain respondent confidentiality. This research was approved by the Louisiana Department of Health and LSU Health Sciences Center’s Institutional Review Board. All participants received relevant materials, information, and referrals to services or programs as needed following the interview.

### Measures

Age, a calculated variable, was categorized 18–29, 30–39, 40–49, and 50+. Race/Ethnicity was dichotomized as black, Latino, other and white. Household income in the past 12 months included four categories ranging from less than $15,000 to more than $50,000. Education level was categorized less than high school, high school equivalent, some college, and college graduate. Other questions from the NHBS core survey included, sexual identity (gay, straight, or bisexual), current health insurance, and self-reported HIV status.

The CF questions were preceded by the statement ***“****Now I’m going to ask you some questions about belonging to a gay family*, *pageant family*, *or house ball community*. *I will refer to membership in any of these groups as a ‘gay family’*.*”* The CF questions explored participants’ role in the family, number of years they were members, how many of their family members live in other states and how many live in New Orleans. This series questions also sought to understand the proportions of CF members who lived in New Orleans in terms of gender, race, health behaviors, and social capital. Social capital questions were specific to CF members who lived in New Orleans.

The social capital scale developed for this study was based on an extensive literature review to identify a framework for social capital within egocentric social networks. Rationale for the measures included were based upon measures presented by Lakon, Godette and Hipp in the text *Social Capital and Health*[33] who proposed that researchers should consider measuring variations in resources allotted by the network structure from the perspective of ego. We established measures of constructed family-based social capital derived from common indicators of social cohesion and egocentric network properties. No cognitive testing or piloting was done on survey items; however, every effort was made to ask questions about terminology to participants in formative open-ended interviews and all members of the project team provided expert feedback on the final local questionnaire.

The questions primarily embody functional network measures that describe the roles and resources acquired through membership in a group. The eight measures of network properties which were operationalized as social capital for this instrument were derived from the following indicators of social capital: social influence, tie strength (multiplex ties and quality of support), heterogeneity, trust, social cohesion, and compositional quality (advice about general and sexual health). These series of questions were asked in reference to the number of people within each individual participant’s constructed family. Each question began with the phrase, “*Out of all your [constructed] family members who live in New Orleans*, *about how many*…” followed by the indicators of interest. For example, social influence was measured by asking “*Out of all your [constructed] family members who live in New Orleans*, *about how many have influenced important decisions you made in the past three months*?” whereas trust was measured by asking “*Out of all your [constructed] family members who live in New Orleans*, *about how many do you trust in general*?*”* Thus, each participant provided a count in response to social capital items for the number of people in their individual CF. Using the number of each participants’ CF members who live in New Orleans as the denominator, a series of proportions were assessed for each item. The proportions of CF members who fulfilled each measure of social capital for each participant were then averaged across the sample. Verbatim questions are listed in Table 1.

**Table 1:** Eight indicators of social capital within constructed family networks of GBM.

“Out of all your [constructed] family members who live in New Orleans, about how many…”	N	Average of Proportion	Std Dev
**Social Influence**	116	0.40	0.39
have *influenced important decisions you made in the past 3 months*			
**Tie strength (Multiplexity)**	117	0.36	0.39
*fulfill multiple roles in your life (for example*, *they are a member of your* **[constructed]***family but also a co-worker*, *friend*, *or partner*, *etc*.*)*			
**Heterogeneity**	116	0.70	0.35
are *similar to you (in terms of race*, *gender*, *etc*.*)*			
**Trust**	117	0.62	0.39
*do you trust in general*			
**Tie strength (Quality of support)**	117	0.61	0.39
*offer you social support (for example you could talk to them about things that are very personal and private*, *go to them for advice*, *or borrow some money or something valuable if you needed it)*			
Social Cohesion*	113	0.85	0.31
*would not take advantage of you if they got the chance*			
**Compositional Quality**	115	0.65	0.38
*could you ask for advice or help about your health in general*			
**Compositional Quality**	116	0.60	0.41
*could you ask for advice or help about HIV or other STDs*			

*This item was reverse scored.

### Analyses

Survey data were analyzed with SAS 9.3. Basic descriptive statistics of demographic variables and each of the items in the scale (i.e. frequencies, means and standard deviations) were derived using PROC FREQ and PROC MEANS. Bivariate associations between the final proportions for each item on the CF social capital scale and demographic variables were assessed using PROC GLM.

PROC CORR with ALPHA option was used to gauge the reliability of the social capital scale by the Pearson correlation coefficients. The correlation coefficient estimates the degrees of equivalence between all possible ways of splitting and comparing sets of questions for summary scales to ensure items consistently yield similar responses[51]. A priori it was decided that if the alpha for the overall scale increased after removing a particular item then it would be deleted, however if the alpha remained unchanged or decreased after removing an item, the item would be retained.

PROC FACTOR was used to assess the dimensionality of the scales. Initial factor analyses, followed by varimax rotation, were used to examine the following criteria for factors: eigenvalue, the scree test, and percent of variance extracted. The results of the factor analysis (i.e. the scree plot and eigenvalues) were used to interpret the number of factors and factor loadings to determine construct validity. The measure for social cohesion (whether or not network members would take advantage of a participant) was reverse scored to align all the measures in the same direction. Thus, the proportion presented in the results reflects the average number of CF members who would not take advantage of the participant if given the chance.

## Results and discussion

Each measure and item are shown in Table 1 with the average proportions of social capital responses by participants. On average, participants reported that 10 members of their constructed families had influenced important decisions in the past month and the average size of constructed families was approximately 20 individuals (not shown). Thus, respondents depended on approximately 40% of their CF members to help them make important life decisions. The multiplicity of social ties within families was also apparent: 36% of constructed families fulfilled multiple roles in a person’s life. There were high levels of heterogeneity such that 70% of CF members were similar to the participant in terms of race, gender or other factors. Participants also reportedly trusted the majority of their CF members on average (60%) and felt comfortable asking for help or advice about health issues (65%) and HIV or other STDs (60%). Thus, relatively high levels for each social capital measure were reported.

The measures were modestly correlated with one another (see Table 2), with the exception of heterogeneity and social cohesion. The Cronbach Coefficient Alpha for the correlation of the scale using all eight variables was 0.77. When heterogeneity was removed from the model, the alpha increased to .80. Upon removing both heterogeneity and the social cohesion variable from the correlation matrix the alpha increased to 0.84.

**Table 2:** Correlations between CF-provided social capital measures.

Measure	*1*	*2*	*3*	*4*	*5*	*6*	*7*	*8*
Social Influence	1							
	116							
Multiplex Ties	0.41***	1						
	116	117						
Heterogeneity	0.035	0.08	1					
	115	116	116					
Trust	0.34**	0.39***	0.11	1				
	116	117	116	117				
Social Cohesion	-0.01	0.04	0.19*	0.20*	1			
	112	113	112	113	113			
Quality of Support	0.42***	0.38***	-0.03	0.52***	0.01	1		
	116	117	116	117	113	117		
Compositional Quality (Health)	0.42***	0.46***	0.13	0.60***	0.27**	0.58***	1	
	114	115	114	115	111	115	115	
Compositional Quality (HIV)	0.28**	0.41***	0.18	0.66***	0.23*	0.45***	0.60***	1
	115	116	115	116	112	116	114	116

*P < .05.

**P < .01.

***P < .0001.

Note: Number of observations displayed below *r*

After assessing the correlation coefficient alpha to estimate the degrees of equivalence between all possible ways of comparing a summary scale to ensure similar responses are consistently obtained, a subsequent factor analysis of all eight measures led to the removal of social cohesion and heterogeneity. Table 3 shows the results of the factor analysis assessing all eight measures of social capital provided by constructed families.

**Table 3:** Factor loadings for eight CF-provided social capital measures.

	Default		Varimax Rotation		Final Solution
	Factor 1	Factor 2	Factor 1	Factor 2	Factor 1
Social Influence	0.58	-0.37	0.66	-0.20	0.62
Multiplex Ties	0.64	-0.19	0.67	-0.01	0.66
Heterogeneity	0.18	0.66	-0.01	0.68	-
Trust	0.81	0.01	0.78	0.23	0.80
Quality of Support	0.74	-0.30	0.79	-0.09	0.75
Social Cohesion	0.28	0.72	0.07	0.77	-
Compositional Quality- Health	0.84	0.09	0.78	0.32	0.83
Compositional Quality- HIV	0.79	0.19	0.71	0.40	0.78

The Eigenvalue of 3.39 for the first factor followed by 1.26 for the second factor in addition to the scree plot indicated a one factor solution. A confirmatory factor analysis excluded heterogeneity and social cohesion. A final single factor solution was obtained which was comprised of social influence, multiplex ties, trust, tie strength, and two measures of compositional quality with high factor loadings. The final Eigenvalue after both measures were removed was 3.31 and no other measures had Eigenvalues above 1 in the final solution. The resulting measures were highly correlated with an alpha of 0.84 and each factor loading was well above 0.3.

The final scale was operationalized as a score by summing the average of the proportion for each measure and dividing by six (i.e. the final number of measures from factor analyses). The final score ranged from zero to one with one referring to a higher social capital and zero indicating a lower social capital score. The measure had a normal distribution with the highest observation of 1 and the lowest value at 0.009.

The bivariate associations with the final CF-provided social capital scores are shown in Table 4. In total, 131 GBM completed questions about social capital within their constructed families. The majority of participants were black, Latino, or other race (59%), under 30 years of age, and identified as gay. Sixty-six percent had health insurance and 76% were HIV negative at the time of the interview. Nearly half of the sample reported household incomes below $30,000 annually. The majority of the sample (69%) had at least some college education. GBM who belonged to constructed families without family names had higher social capital than those in named families (p = 0.0284). Thus, our hypothesis that members of constructed families with names might have higher social capital was not upheld. On average, GBM in named constructed families had a lower social capital score than those in unnamed families (*β* = -0.12).

**Table 4:** Bivariate associations with CF-provided social capital scores.

	N = 131	%	Mean	β	*p-*value
**Constructed Family**					**0.0284**
* Named*	73	56%	0.49	-0.12	
No Name*	58	44%	0.61		
**Age**					**0.6225**
* 18–29*	67	51%	0.51	-0.07	
* 30–39*	33	25%	0.55	-0.03	
* 40–49*	9	7%	0.62	0.04	
50+*	22	17%	0.58		
**Race**					**0.1070**
* White*	54	41%	0.50	0.09	
* Black*, *Latino*, Other Race*	77	59%	0.59		
**Education**					**0.3058**
* College Grad*	42	32%	0.61	0.24	
* Some College*	49	37%	0.51	0.14	
* High School Grad*	37	28%	0.52	0.15	
Less High School*	3	2%	0.37		
**Income**					**0.2423**
* Less than $15*,*000*	39	30%	0.54	-0.03	
* $15*,*000 - $29*,*999*	35	27%	0.58	0.01	
* $30*,*000 - $49*,*999*	20	16%	0.41	-0.16	
* $50*,000 +*	35	27%	0.57		
**Sexual Identity**					0.3977
* Gay*	113	86%	0.54	-0.13	
* Bisexual*	15	12%	0.45	-0.23	
Heterosexual*	3	2%	0.68		
**Health Insurance**					**0.2683**
* Yes*	86	66%	0.56	0.06	
No*	45	34%	0.49		
**HIV Status**				**-0.07**	
* Negative*	100	76%	0.59		0.2535
Positive*	31	24%	0.52		

*Denotes referent group

## Conclusions

The novel scale presented here includes six indicators of social capital at the network level to discern social capital attributed to membership in constructed families of GBM. No such tool has previously been developed for this population in the US or internationally. Previous research among GBM in the US context has yet to examine social capital as it pertains to social group memberships, including constructed families. The only previous study which investigates social capital specific to GBM participation in subgroups took place in Swaziland[43]. Grover and colleagues employed a modified social cohesion scale among GBM in South Africa based upon previous work among female sex workers[52]. This scale measured participation using an index comprised of four questions regarding membership or attendance in church, clubs, cultural activities, and other community activities. Social cohesion was measured by asking GBM the degree to which they felt they could count on other GBM for resources, trust, social integration, and disagreements. Our study differs from previous approaches because it incorporates measures of egocentric network properties and social cohesion to measure social capital among CF of GBM.

This scale is a unique contribution to the social capital literature because we intertwine social cohesion within the context of constructed family networks of GBM and additionally may be relevant in other populations to examine both social and sexual network connections. The CF social capital scale may also be useful for regression analyses to demonstrate how different levels of social capital conferred by membership in certain networks influence health outcomes. Young GBM of color are frequently discussed in the public health literature as being “at risk” or “vulnerable” to poor health outcomes due to the resource-poor social contexts of their lives. The creation of this scale allows for the empirical documentation of endogenous forms of support fostered by young GBM in constructed families. For example, social cohesion could result in community empowerment among GBM which may improve HIV prevention and care for GBM. Future studies should investigate this scale in relation to HIV risk and prevention behaviors among GBM and other populations including transgender women.

Social capital measures may be used to study health outcomes and inform services and programs that seek to harness existing networks or policies designed to foster strength and resilience within marginalized LGBT populations. Such approaches are important to future research and interventions in both pragmatic and ethical terms. Interventions may be most effective and efficient when building on existing social connections, and this scale could be used to identify within- and across-group variations in social capital. Thus, this scale may aid in the development of strategies which access inherent opportunities to mobilize the LGBT community to promote healthy behaviors. Relatedly, the use of *intraventions*, (i.e. interventions formed by and within specific populations), have been described as critical opportunities for HIV prevention for GBM within the house and ball community[53]. Similar approaches may be useful for the wider inclusion of other forms of constructed families which are meaningful support systems in the lives of gay and bisexual men and transgender women.

Limitations of this study should be noted. First, the sample size for this study was constrained by membership in constructed families. Because GBM belong to a number of social or sexual networks, a larger contribution to the field will be to modify this scale for use within other social and sexual networks to assess whether the instrument exhibited the same factor structure. The results of this study are drawn from cross-sectional data from a single demographic surveillance site at one point in time and therefore do not include test-retest reliability. This scale has not been independently validated by a group of GBM who did not participate in the research. Therefore, this scale may be reliable but not valid. While this study analyzed network-level social capital among individuals, the use of individual-level questions asked about a network or group level property may be an additional limitation. Furthermore, these items have not been correlated with other social capital measures. Nevertheless, this newly developed social capital scale increases the public health understanding of resources provided to GBM through membership in constructed families.

This study also contributes to literature surrounding the formation of families beyond traditional biological and legal ties, and broadens definitions of family among GBM, particularly GBM of color. We provide a new conceptualization of social capital derived from chosen kinship groups to which GBM belong. As the wider examination within social science literature surrounding social capital has illustrated, new conceptualizations of social capital should incorporate network properties. It has been argued that it is easier to measure the byproducts, outcomes, causes or consequences of social capital and a number of researchers have lost sight of what is being measured[54]. This social capital scale was shown to be a reliable measure of six dimensions of constructed family networks. This tool may be useful for conceptualizing social capital provided from families or other social organizations, or for use within interventions harnessing networks of GBM to encourage testing, treating, linking and retaining network members in HIV care.

## References

[1] Centers for Disease Control and Prevention. HIV among Gay and Bisexual Men [Internet]. CDC Fact Sheet. 2017 [2018 May 22]. https://www.cdc.gov/nchhstp/newsroom/docs/factsheets/cdc-msm-508.pdf

[2] CDC. Lifetime Risk of HIV Diagnosis. CROI Press Release. 2016 [2018 May 22]; https://www.cdc.gov/nchhstp/newsroom/2016/croi-press-release-risk.html

[3] Holloway IW, Traube DE, Kubicek K, Supan J, Weiss G, Kipke MD. HIV prevention service utilization in the Los Angeles House and Ball communities: past experiences and recommendations for the future. AIDS Educ Prev. 2012 10 [2018 May 22];24(5):431–44. Available from: http://guilfordjournals.com/doi/10.1521/aeap.2012.24.5.431 10.1521/aeap.2012.24.5.431 23016504 10.1521/aeap.2012.24.5.431 PMC3507445

[4] Sanchez T, Finlayson T, Murrill C, Guilin V, Dean L. Risk behaviors and psychosocial stressors in the new york city house ball community: a comparison of men and transgender women who have sex with men. AIDS Behav. 2010 4 10 [2017 Nov 21];14(2):351–8. Available from: http://link.springer.com/10.1007/s10461-009-9610-6 10.1007/s10461-009-9610-6 19763812 10.1007/s10461-009-9610-6

[5] Murrill CS, Liu K-L, Guilin V, Colón ER, Dean L, Buckley LA, et al HIV prevalence and associated risk behaviors in New York City’s house ball community. Am J Public Health. 2008 6 [2018 May 22];98(6):1074–80. Available from: http://www.ncbi.nlm.nih.gov/pubmed/18445806 10.2105/AJPH.2006.108936 18445806 10.2105/AJPH.2006.108936 PMC2377289

[6] Zarwell M. Subgroups of men who have sex with men, social capital, and HIV risk behaviors. Dissertation. 2016 [2017 Nov 21]; https://search.proquest.com/openview/aedc7d6b22498c95afb3b3c38f5907df/1.pdf?pq-origsite=gscholar&cbl=18750&diss=y

[7] Zarwell MC, Robinson WT. The Influence of Constructed Family Membership on HIV Risk Behaviors among Gay, Bisexual, and Other Men Who Have Sex with Men in New Orleans. J Urban Heal. 2017 10 18 [2017 Nov 21]; Available from: http://www.ncbi.nlm.nih.gov/pubmed/29047021 10.1007/s11524-017-0203-9 PMC5906379 29047021

[8] Arnold EA, Bailey MM. Constructing Home and Family: How the Ballroom Community Supports African American GLBTQ Youth in the Face of HIV/AIDS. J Gay Lesbian Soc Serv. 2009 5 20 [2017 Nov 21];21(2–3):171–88. Available from: http://www.tandfonline.com/doi/abs/10.1080/10538720902772006 10.1080/10538720902772006 23136464 10.1080/10538720902772006 PMC3489283

[9] Arnold EA, Sterrett-Hong E, Jonas A, Pollack LM. Social networks and social support among ball-attending African American men who have sex with men and transgender women are associated with HIV-related outcomes. Glob Public Health. 2018 2 11 [2017 Dec 19];13(2):144–58. Available from: http://www.ncbi.nlm.nih.gov/pubmed/27169632 10.1080/17441692.2016.1180702 27169632 10.1080/17441692.2016.1180702 PMC5106335

[10] Schrager SM, Latkin CA, Weiss G, Kubicek K, Kipke MD. High-Risk Sexual Activity in the House and Ball Community: Influence of Social Networks. Am J Public Health. 2014 2 [2017 Dec 19];104(2):326–31. Available from: http://www.ncbi.nlm.nih.gov/pubmed/24328654 10.2105/AJPH.2013.301543 24328654 10.2105/AJPH.2013.301543 PMC3935685

[11] Kipke MD, Kubicek K, Supan J, Weiss G, Schrager S. Laying the groundwork for an HIV prevention intervention: a descriptive profile of the Los Angeles House and Ball communities. AIDS Behav. 2013 3 15 [2017 Nov 21];17(3):1068–81. Available from: http://link.springer.com/10.1007/s10461-012-0227-9 10.1007/s10461-012-0227-9 22699855 10.1007/s10461-012-0227-9 PMC3492531

[12] Kubicek K, Beyer WH, McNeeley M, Weiss G, Omni LFTU, Kipke MD. Community-engaged research to identify house parent perspectives on support and risk within the House and Ball scene. J Sex Res. 2013 2 [2017 Nov 21];50(2):178–89. Available from: http://www.tandfonline.com/doi/abs/10.1080/00224499.2011.637248 10.1080/00224499.2011.637248 22206442 10.1080/00224499.2011.637248 PMC3432658

[13] Oswald RF. Resilience Within the Family Networks of Lesbians and Gay Men: Intentionality and Redefinition. J Marriage Fam. 2002 5 1 [2017 Nov 21];64(2):374–83. Available from: http://doi.wiley.com/10.1111/j.1741-3737.2002.00374.x

[14] Horne SG, Levitt HM, Sweeney KK, Puckett JA, Hampton ML. African American Gay Family Networks: An Entry Point for HIV Prevention. J Sex Res. 2015 9 2 [2017 Nov 21];52(7):807–20. Available from: http://www.tandfonline.com/doi/full/10.1080/00224499.2014.901285 10.1080/00224499.2014.901285 24992185 10.1080/00224499.2014.901285

[15] Levitt HM, Sharon Horne BG, Darren Freeman-Coppadge B, Tangela Roberts B. HIV Prevention in Gay Family and House Networks: Fostering Self-Determination and Sexual Safety. [2018 5 17]; Available from: https://link.springer.com/content/pdf/10.1007%2Fs10461-017-1774-x.pdf 10.1007/s10461-017-1774-x 28451890

[16] Weston Kath. Families We Choose: Lesbians, Gays, and Kinship. New York, NY: Columbia University Press; 1991.

[17] Lewin E. Lesbian and Gay Kinship: Kath Weston’s &quot;Families We Choose&quot; and Contemporary Anthropology [Internet]. Vol. 18, Signs. The University of Chicago Press; [2017 Nov 21]. p. 974–9. https://www.jstor.org/stable/3174919

[18] Dickson-Gomez J, Owczarzak J, St Lawrence J, Sitzler C, Quinn K, Pearson B, et al Beyond the ball: implications for HIV risk and prevention among the constructed families of African American men who have sex with men. AIDS Behav. 2014 11 1 [2017 Nov 21];18(11):2156–68. Available from: http://link.springer.com/10.1007/s10461-014-0836-6 10.1007/s10461-014-0836-6 24980248 10.1007/s10461-014-0836-6 PMC4198443

[19] Young LE, Jonas AB, Michaels S, Jackson JD, Pierce ML, Schneider JA, et al Social-structural properties and HIV prevention among young men who have sex with men in the ballroom house and independent gay family communities. Soc Sci Med. 2017 2 [2017 Nov 21];174:26–34. Available from: http://www.ncbi.nlm.nih.gov/pubmed/27987435 10.1016/j.socscimed.2016.12.009 27987435 10.1016/j.socscimed.2016.12.009 PMC5258653

[20] Kawachi I, Kim D, Coutts A, Subramanian S V. Commentary: Reconciling the three accounts of social capital. Int J Epidemiol. 2004 7 28 [2018 May 22];33(4):682–90. Available from: http://www.ncbi.nlm.nih.gov/pubmed/15282222 10.1093/ije/dyh177 15282222 10.1093/ije/dyh177

[21] Baum FE, Ziersch AM. Social capital. J Epidemiol Community Health. 2003 5 [2018 May 22];57(5):320–3. Available from: http://www.ncbi.nlm.nih.gov/pubmed/12700212 10.1136/jech.57.5.320 12700212 10.1136/jech.57.5.320 PMC1732452

[22] Campbell C, Mzaidume Y. How can HIV be prevented in South Africa? A social perspective. BMJ. 2002 1 26 [2018 May 22];324(7331):229–32. Available from: http://www.ncbi.nlm.nih.gov/pubmed/11809649 11809649 10.1136/bmj.324.7331.229 PMC1122138

[23] Kirst MJ. Social Capital and Beyond: A Qualitative Analysis of Social Contextual and Structural Influences on Drug-Use Related Health Behaviors. J Drug Issues. 2009 7 1 [2017 Dec 19];39(3):653–76. Available from: http://journals.sagepub.com/doi/10.1177/002204260903900309

[24] Friedman SR, Mateu-Gelabert P, Curtis R, Maslow C, Bolyard M, Sandoval M, et al Social capital or networks, negotiations, and norms? A neighborhood case study. Am J Prev Med. 2007 6 [2018 May 22];32(6 Suppl):S160–70. Available from: http://www.ncbi.nlm.nih.gov/pubmed/17543707 10.1016/j.amepre.2007.02.005 17543707 10.1016/j.amepre.2007.02.005 PMC1995560

[25] Carpiano RM. Toward a neighborhood resource-based theory of social capital for health: Can Bourdieu and sociology help? Soc Sci Med. 2006 1 1 [2018 May 22];62(1):165–75. Available from: https://www.sciencedirect.com/science/article/pii/S0277953605002546 10.1016/j.socscimed.2005.05.020 15992978 10.1016/j.socscimed.2005.05.020

[26] Kawachi I, Subramanian SV. Social epidemiology for the 21st century. Soc Sci Med. 2018 1 [2018 May 22];196:240–5. Available from: http://www.ncbi.nlm.nih.gov/pubmed/29113687 10.1016/j.socscimed.2017.10.034 29113687 10.1016/j.socscimed.2017.10.034

[27] Bourdieu P. The Forms of Capital In: Richardson J, editor. Handbook of Theory and Research for the Sociology of Education. Westport, CT: Greenwood; 1986 [2018 May 22]. p. 241–58. http://home.iitk.ac.in/~amman/soc748/bourdieu_forms_of_capital.pdf

[28] Lin N. Building a Network Theory of Social Capital. Connections. 1999 [2018 May 24];22(1):28–51. Available from: http://www.insna.org/PDF/Keynote/1999.pdf

[29] Van Der Gaag M.; Snijders T. The Resource Generator: social capital quantification with concrete items. Soc Networks. 2005 [2018 May 24];27:1–29. http://citeseerx.ist.psu.edu/viewdoc/download?doi=10.1.1.475.8064&rep=rep1&type=pdf

[30] Van Der Gaag M, Webber M. Measurement of Individual Social Capital In: Social Capital and Health. New York, NY: Springer New York; 2008 [2018 May 22]. p. 29–49. http://link.springer.com/10.1007/978-0-387-71311-3_2

[31] Robert D. Putnam Bowling Alone [Internet]. 1st ed Princeton, NJ: Princeton University Press; 2000 [2018 May 22]. http://bowlingalone.com/

[32] Burt RS. The Network Structure Of Social Capital. Res Organ Behav. 2000 1 1 [2018 May 22];22:345–423. Available from: https://www.sciencedirect.com/science/article/pii/S0191308500220091

[33] Lakon C, Godette D, H J. Network-based approaches for measuring social capital In: Kawachi I, Subramanian SV KD, editor. Social capital and Health. New York, New York: Springer; 2010 p. 63.

[34] Lovell AM. Risking risk: the influence of types of capital and social networks on the injection practices of drug users. Soc Sci Med. 2002 9 [2017 Dec 19];55(5):803–21. Available from: http://www.ncbi.nlm.nih.gov/pubmed/12190272 12190272 10.1016/s0277-9536(01)00204-0

[35] Phillips JC, Webel A, Rose CD, Corless IB, Sullivan KM, Voss J, et al Associations between the legal context of HIV, perceived social capital, and HIV antiretroviral adherence in North America. BMC Public Health. 2013 12 8 [2018 May 16];13(1):736 Available from: http://www.ncbi.nlm.nih.gov/pubmed/23924399 23924399 10.1186/1471-2458-13-736 PMC3750916

[36] Ransome Y, Galea S, Pabayo R, Kawachi I, Braunstein S, Nash D. Social Capital is Associated With Late HIV Diagnosis. JAIDS J Acquir Immune Defic Syndr. 2016 10 1 [2018 May 16];73(2):213–21. Available from: http://www.ncbi.nlm.nih.gov/pubmed/27632146 10.1097/QAI.0000000000001043 27632146 10.1097/QAI.0000000000001043 PMC5026389

[37] Holtgrave DR, Crosby RA. Social capital, poverty, and income inequality as predictors of gonorrhoea, syphilis, chlamydia and AIDS case rates in the United States. Sex Transm Infect. 2003 2 [2018 May 22];79(1):62–4. Available from: http://www.ncbi.nlm.nih.gov/pubmed/12576618 10.1136/sti.79.1.62 12576618 10.1136/sti.79.1.62 PMC1744600

[38] Pronyk PM, Harpham T, Morison LA, Hargreaves JR, Kim JC, Phetla G, et al Is social capital associated with HIV risk in rural South Africa? Soc Sci Med. 2008 5 [2018 May 16];66(9):1999–2010. Available from: http://www.ncbi.nlm.nih.gov/pubmed/18299168 10.1016/j.socscimed.2008.01.023 18299168 10.1016/j.socscimed.2008.01.023

[39] Frumence G, Killewo J, Kwesigabo G, Nyström L, Eriksson M, Emmelin M. Social capital and the decline in HIV transmission—A case study in three villages in the Kagera region of Tanzania. SAHARA J J Soc Asp HIV/AIDS Res Alliance. 2010 10 [2018 May 16];7(3):9–20. Available from: http://www.ncbi.nlm.nih.gov/pubmed/21409300 10.1080/17290376.2010.9724964 21409300

[40] Moore S, Haines V, Hawe P, Shiell A. Lost in translation: a genealogy of the &quot;social capital&quot; concept in public health. J Epidemiol Community Heal. 2006 8 1 [2018 May 22];60(8):729–34. Available from: http://www.ncbi.nlm.nih.gov/pubmed/16840764 10.1136/jech.2005.041848 PMC2588078 16840764

[41] Bettinger JA, Adler NE, Curriero FC, Ellen JM. Risk perceptions, condom use, and sexually transmitted diseases among adolescent females according to social network position. Sex Transm Dis. 2004 9 [2018 May 22];31(9):575–9. Available from: http://www.ncbi.nlm.nih.gov/pubmed/15480121 15480121 10.1097/01.olq.0000137906.01779.55

[42] Gregson S, Mushati P, Grusin H, Nhamo M, Schumacher C, Skovdal M, et al Social capital and women’s reduced vulnerability to HIV infection in rural Zimbabwe. Popul Dev Rev. 2011 [2018 May 22];37(2):333–59. Available from: http://www.ncbi.nlm.nih.gov/pubmed/22066129 22066129 10.1111/j.1728-4457.2011.00413.x PMC3302682

[43] Grover E, Grosso A, Ketende S, Kennedy C, Fonner V, Adams D, et al Social cohesion, social participation and HIV testing among men who have sex with men in Swaziland. AIDS Care. 2016 6 2 [2018 May 16];28(6):795–804. Available from: http://www.tandfonline.com/doi/full/10.1080/09540121.2015.1131971 10.1080/09540121.2015.1131971 26824888 10.1080/09540121.2015.1131971

[44] Berkman LF, Glass T, Brissette I, Seeman TE. From social integration to health: Durkheim in the new millennium. Soc Sci Med. 2000 9 [2018 May 22];51(6):843–57. Available from: http://www.ncbi.nlm.nih.gov/pubmed/10972429 10972429 10.1016/s0277-9536(00)00065-4

[45] Berkman LF. Social Epidemiology: Social Determinants of Health in the United States: Are We Losing Ground? Annu Rev Public Health. 2009 4 [2017 Nov 21];30(1):27–41. Available from: http://www.ncbi.nlm.nih.gov/pubmed/19705554 19705554 10.1146/annurev.publhealth.031308.100310

[46] Adler PS, Kwon S-W. Social Capital: Prospects for a New Concept. Acad Manag Rev. 2002 1 [2018 May 22];27(1):17 Available from: http://links.jstor.org/sici?sici=0363-7425%28200201%2927%3A1%3C17%3ASCPFAN%3E2.0.CO%3B2-5&origin=crossref

[47] Kim D, Subramanian SV, Kawachi I. Social Capital and Physical Health In: Social Capital and Health. New York, NY: Springer New York; 2008 [2018 May 22]. p. 139–90. http://link.springer.com/10.1007/978-0-387-71311-3_8

[48] Kawachi I, Subramanian SV, Kim D. Social Capital and Health In: Social Capital and Health. New York, NY: Springer New York; 2008 [2018 May 22]. p. 1–26. http://link.springer.com/10.1007/978-0-387-71311-3_1

[49] Lansky A, Sullivan PS, Gallagher KM, Fleming PL. HIV Behavioral Surveillance in the U.S.: A Conceptual Framework. Public Health Rep. 2007 1 2 [2018 May 22];122(1_suppl):16–23. Available from: http://www.ncbi.nlm.nih.gov/pubmed/17354523 17354523 10.1177/00333549071220S104 PMC1804114

[50] MacKellar DA, Gallagher KM, Finlayson T, Sanchez T, Lansky A, Sullivan PS. Surveillance of HIV Risk and Prevention Behaviors of Men Who Have Sex with Men—A National Application of Venue-Based, Time-Space Sampling. Public Health Rep. 2007 1 2 [2018 May 22];122(1_suppl):39–47. Available from: http://www.ncbi.nlm.nih.gov/pubmed/17354526 17354526 10.1177/00333549071220S107 PMC1804106

[51] Aday LA, Cornelius LJ. Designing and Conducting Health Surveys A Comprehensive Guide [Internet]. 3rd ed San Francisco, CA: John Wiley & Sons; 2006 [2018 May 16]. 546 p. https://www.wiley.com/en-us/Designing+and+Conducting+Health+Surveys%3A+A+Comprehensive+Guide%2C+3rd+Edition-p-9781118046678

[52] Fonner VA, Kerrigan D, Mnisi Z, Ketende S, Kennedy CE, Baral S. Social Cohesion, Social Participation, and HIV Related Risk among Female Sex Workers in Swaziland. Sueur C, editor. PLoS One. 2014 1 31 [2018 May 25];9(1):e87527 Available from: http://dx.plos.org/10.1371/journal.pone.0087527 24498125 10.1371/journal.pone.0087527 PMC3909117

[53] Bailey MM. Performance as Intravention: Ballroom Culture and the Politics of HIV/AIDS in Detroit. Souls. 2009 9 8 [2018 May 22];11(3):253–74. Available from: http://www.tandfonline.com/doi/abs/10.1080/10999940903088226

[54] Appel L, Dadlani P, Dwyer M, Hampton K, Kitzie V, Matni ZA, et al Testing the validity of social capital measures in the study of information and communication technologies. Information, Commun Soc. 2014 4 21 [2018 May 22];17(4):398–416. Available from: http://www.tandfonline.com/doi/abs/10.1080/1369118X.2014.884612

